# SKF83959, an Agonist of Phosphatidylinositol-Linked D_1_-Like Receptors, Promotes ERK1/2 Activation and Cell Migration in Cultured Rat Astrocytes

**DOI:** 10.1371/journal.pone.0049954

**Published:** 2012-11-19

**Authors:** Chao Huang, Jingjing Wu, Rujia Liao, Wei Zhang

**Affiliations:** 1 Department of Pharmacology, School of Medicine, Nantong University, Nantong, Jiangsu, People’s Republic of China; 2 Department of Internal Medicine, Suzhou Kowloon Hospital, Suzhou, Jiangsu, People’s Republic of China; Albany Medical College, United States of America

## Abstract

Extracellular signal-regulated kinase 1/2 (ERK1/2) is a member of the mitogen-activated protein kinase family. It can mediate cell migration. Classical dopamine receptor-mediated ERK1/2 phosphorylation is widely studied in neurons. Here, we report that ERK1/2 phosphorylation is also modulated by putative phosphatidylinositol-linked D_1_-like receptors in cultured rat astrocytes. 6-chloro-7,8-dihydroxy-3-methyl-1-(3-methylphenyl)-2,3,4,5-tetrahydro-1H-3-benzazepine (SKF83959), an agonist of the putative phosphatidylinositol-linked D_1_-like receptors, was found to enhance ERK1/2 phosphorylation, which then promoted the migration of cultured astrocytes. The SKF83959-induced ERK1/2 phosphorylation was found to be Ca^2+^-independent based on the following observations: i. chelating intracellular Ca^2+^ did not inhibit ERK1/2 phosphorylation and astrocyte migration; ii. blockage of the release of intracellular Ca^2+^ from the endoplasmic reticulum by an inhibitor of inositol 1,4,5-trisphosphate (IP3) receptor did not attenuate ERK1/2 phosphorylation. However, inhibition of phospholipase C (PLC), the upstream molecule of internal Ca^2+^ release, disabled SKF83959’s ability to elevate the level of ERK1/2 phosphorylation. Both non-selective protein kinase C (PKC) inhibitor and PKCδ selective inhibitor prevented ERK1/2 phosphorylation increase and astrocyte migration, but PKCα inhibitor did not. This suggests that Ca^2+^-independent and diacylglycerol-dependent PKCδ acts downstream of putative phosphatidylinositol-linked D_1_-like receptor activation and mediates SKF83959-induced elevation of ERK1/2 phosphorylation in order to modulate astrocyte migration. In conclusion, our results demonstrate that SKF83959-induced increases in ERK1/2 phosphorylation and astrocyte migration are dependent on PLC-PKCδ signals. This might help us to further understand the functions of the putative phosphatidylinositol-linked D_1_-like receptors in the nervous system.

## Introduction

Dopamine (DA) can regulate emotion, cognition, locomotion, and endocrine function [1], [2]. The roles of DA are mediated by distinct DA receptors (D_1_–D_5_). Among these receptors, classical cyclase-coupled D_1_ receptors are linked to Gs protein that can stimulate cyclic AMP (cAMP) formation [3]. However, non-cyclase-coupled D_1_-like receptors are connected to Gq protein to promote phospholipase C (PLC) activation and the subsequent hydrolysis of phosphatidylinositol 4,5-bisphosphate (PIP2) [4]. Non-cyclase-coupled D_1_-like receptor was named phosphatidylinositol (PI)-linked D_1_-like receptor because of its ability to activate Gq/PLC/inositol 1,4,5-triphosphate (IP3) signals [5]. SKF83959, an agonist of the putative PI-linked D_1_-like receptor can be used to identify new roles of atypical DA receptors in the nervous system [6], [7], [8]. For example, one study showed that stimulation of striatal neurons by SKF83959 induces an inhibition of high-voltage-activated (HVA) Ca^2+^ currents, which was proven to be dependent on PLC/IP3/Ca^2+^/calcineurin signals [9]. SKF83959 can also alleviate dyskinesia, a symptom of Parkinson’s disease, in *in vivo* models [7]. In brain slices, SKF83959 activates the cAMP-response element binding protein (CREB) and dopamine and adenosine 3′5′-monophosphate-regulated phospho-protein 32 (DARPP-32) via PLC/IP3/Ca^2+^/calcium-calmodulin-dependent protein kinase II (CaMKII) and PLC/IP3/Ca^2+^/cyclin-dependent kinase 5 (CDK5) signals, respectively [10].

Astrocytes, regarded as supporting structures in the nervous system, are generally thought to act as a syncytium of interconnected cells, rather than as individual bodies [11]. In most cases, the functions of astrocytes are mediated primarily by their membrane transporters and receptors such as the glutamate transporters and classical DA receptors [12], [13], [14]. Putative PI-linked D_1_-like receptors have also been found to modulate astrocyte function. For instance, activation of the putative PI-linked D_1_-like receptors by SKF83959 up-regulates astrocyte-derived fibroblast growth factor-2 (FGF-2) expression via PLC/IP3/Ca^2+^/CaMKII signals, which potentially protects dopaminergic neurons from 1-Methyl-4-phenyl-1,2,3,6-tetrahydropyridine (MPTP)-induced neurotoxicity [6]. Regardless of whether the targets of SKF83959 are astrocytes or neurons, the known effects of SKF83959 correlate with the increase in intracellular Ca^2+^. Data from our previous study also showed a release of internal Ca^2+^ from endoplasmic reticulum (ER) in cultured astrocytes after SKF83959 treatment [15]. Previous studies mainly evaluated changes in the activation of Ca^2+^-related kinases such as CaMKII in response to SKF83959 application. However, due to the complexity of DA signal transduction pathways, we focused on whether other signal molecules could mediate SKF83959’s effects on astrocytes.

Extracellular signal-regulated kinase 1/2 (ERK1/2) may be involved in this process. ERK1/2 is a member of the mitogen-activated protein kinase family, whose activation in response to stimuli is involved in cell migration and proliferation [16], [17]. For example, activation of the ERK1/2 signals promotes transforming growth factor-β1-induced astrocyte migration [18]. Chronic ERK1/2 activation in neurodegenerative disorders such as Alzheimer’s disease and Parkinson’s disease can be mediated by the classical DA receptors [19]–[21]. However, it is still unclear whether the putative PI-linked D_1_-like receptors can mediate ERK1/2 activation in cultured astrocytes. Our present study demonstrates that SKF83959 promotes ERK1/2 phosphorylation by augmenting PLC-protein kinase Cδ (PKCδ) signaling in cultured rat astrocytes. Both ERK1/2 and PKCδ inhibition functionally inhibit SKF83959-induced astrocyte migration. Our observations regarding SKF83959-induced ERK1/2 activation in astrocytes might provide new perspectives on the roles of the putative PI-linked D_1_-like receptors in the nervous system.

## Materials and Methods

### Chemicals and Reagents

Dulbecco’s modified Eagle’s medium/F12 (DMEM/F12) was obtained from Gibco Invitrogen Corporation (Carlsbad, CA, USA). Heat-inactivated fetal bovine serum was purchased from Hyclone (Logan, UT, USA). Poly-L-lysine, PD98059, BAPTA-AM, GF109203X, Gö6976, prazosin, hydroxyurea, rottlerin, and Hoechst 33258 were purchased from Sigma (Saint Louis, MO, USA). SKF83959, 2-APB, SCH23390, and U-73122 were purchased from Tocris Bioscience (Ellisville, MO, USA). Antibodies against phospho-ERK1/2 and total-ERK1/2 were obtained from Santa Cruz Biotechnology (Santa Cruz, CA, USA) and Abcam (Cambridge, MA, USA), respectively. Antibodies against total-CaMKII, phospho-CaMKII (Thr286), and β-actin were purchased from Cell Signaling Technology (Beverly, MA, USA). Antibody against GFAP was the product of NeoMarkers (Fremont, CA, USA). Both goat anti-mouse IgG polyclonal secondary antibody for Western blot and rabbit anti-mouse tetramethylrhodamine isothiocyanate-conjugated secondary antibody for immunofluorescence were purchased from Thermo Scientific (Rockford, IL, USA). Other related agents were purchased from commercial suppliers. All drugs were prepared as stock solutions, and stock solutions were stored at −20°C. The final concentration of DMSO was < 0.05%. No detectable effects of DMSO were found in our experiments.

### Cell Preparation

The use of rats was approved by the University Animal Ethics Committee of Nantong University (Permit Number: 2110836). Rat primary cultured cells were prepared as described previously with some modifications [22]. Briefly, newborn (day 0–1) rats were decapitated, and their cortex were removed and digested with 0.125% trypsin for 15–20 min at 37°C. Followed by trituration and centrifugation at 118 *g* for 5 min, cells were re-suspended and plated on poly-L-lysine (1 mg/mL)-coated culture flasks. The single-cell suspension was then cultured in DMEM/F12 supplement with 10% heat-inactivated fetal bovine serum and 1% penicillin-streptomycin (100 U/mL). For astrocytes, the medium was changed to fresh DMEM/F12 medium after 24 h and replaced every 3 days. After 12 days, mixed cells were shaken gently overnight (18 h), and then the supernatant was removed. Next the remaining cells were digested with 0.125% trypsin for 5 min and plated on new poly-L-lysine-coated culture flasks. All cultures were maintained in a 37°C incubator containing 95% air and 5% CO_2_. Immunofluorescence with glial fibrillary acidic protein (GFAP) antibody was used to identify the astrocyte. The purity of the astrocyte culture exceeded 98%.

### Immunofluorescence in Rat Astrocytes

Cultured astrocytes were fixed with 4% paraformaldehyde in 0.01 M phosphate-buffered saline (PBS, pH 7.4) for 30 min, and then rinsed three times with PBS for 10 min each. Immunofluorescence study was performed to determine the expression of GFAP in astrocytes. First, cells were permeabilized with PBS containing 0.3% (v/v) Triton X-100 for 30 min and blocked with 1% bovine serum albumin in PBS for 1 h, and then incubated with 1∶100 anti-GFAP antibody in PBS containing 0.3% Triton X-100, 1% bovine serum albumin, and 2% goat serum overnight at 4°C. After rinsing in PBS three times, the cells were incubated with rabbit anti-mouse tetramethylrhodamine isothiocyanate-conjugated secondary antibody (1∶50) for 1 h at room temperature. Then, the cells were incubated with Hoechst 33258 (20 min) for nucleus staining. Eventually, the samples were mounted on glass slides with 30% glycerin and imaged using a confocal laser scanning microscope (FV500; Olympus). The purity of astrocyte culture was calculated by the ratio of the GFAP and Hoechst 33258 co-stained cell numbers to the Hoechst 33258-stained cell numbers.

### Western Blot

To extract the total proteins, purified rat astrocytes were lysed on ice for 30 min in lyses buffer (50 mM Tris-HCl, pH 7.4, 1 mM EDTA, 100 mM NaCl, 20 mM NaF, 3 mM Na_3_VO_4_, 1 mM PMSF, with 1% (v/v) Nonidet P-40, and protease inhibitor cocktail). The lysates were recovered by centrifugation at 12000 *g* for 15 min. After denaturation, the proteins (30 µg) were separated on 10% SDS/PAGE gels and then transferred to nitrocellulose membranes (Bio-Rad, Hercules, CA). After blocking with 5% nonfat dried milk powder/Tris-buffered saline Tween-20 (TBST) for 1 h, membranes were probed with 1∶500 primary antibodies against total ERK1/2 (42/44 kD) and phospho-ERK1/2 (42/44 kD) or 1∶5000 primary antibody against β-actin (43 kD) overnight at 4°C. Primary antibodies were then removed by washing the membranes 3 times in TBST, and incubated for further 1 h at room temperature with goat anti-mouse IgG polyclonal secondary antibody (1∶3000-1∶5000) that are conjugated with polymers of horseradish peroxidase. Following 3 times of washing in TBST, immunoblots were developed on films using the enhanced chemiluminescence (ECL) technique (Pierce, Rockford, USA). The band density was quantified using Image J software. All assays were performed at least three times.

### Cell Migration Assay

Astrocytes plated on poly-L-lysine (1 mg/mL)-coated coverslips were cultured to conﬂuence in 35 mm dishes. The monolayer cells were scratched manually with a pipette tip to create extended and definite scratches in the center of the coverslips with a bright and clear field, and then the detached cells were immediately removed by washing the cells with fresh medium. Growth medium with or without SKF83959 was added to each dish after pretreatment with inhibitors for 30 min. Hydroxyurea, an inhibitor of DNA synthesis [23], was added to prevent the proliferation of astrocytes during the period of observation. Numbers of migratory astrocytes from the scratched boundary were counted from the resulting four phase images for each point with a digital camera and a light microscope (Olympus, Japan) and then averaged for each experimental condition. The data presented were generated from three separate assays.

**Figure 1:**
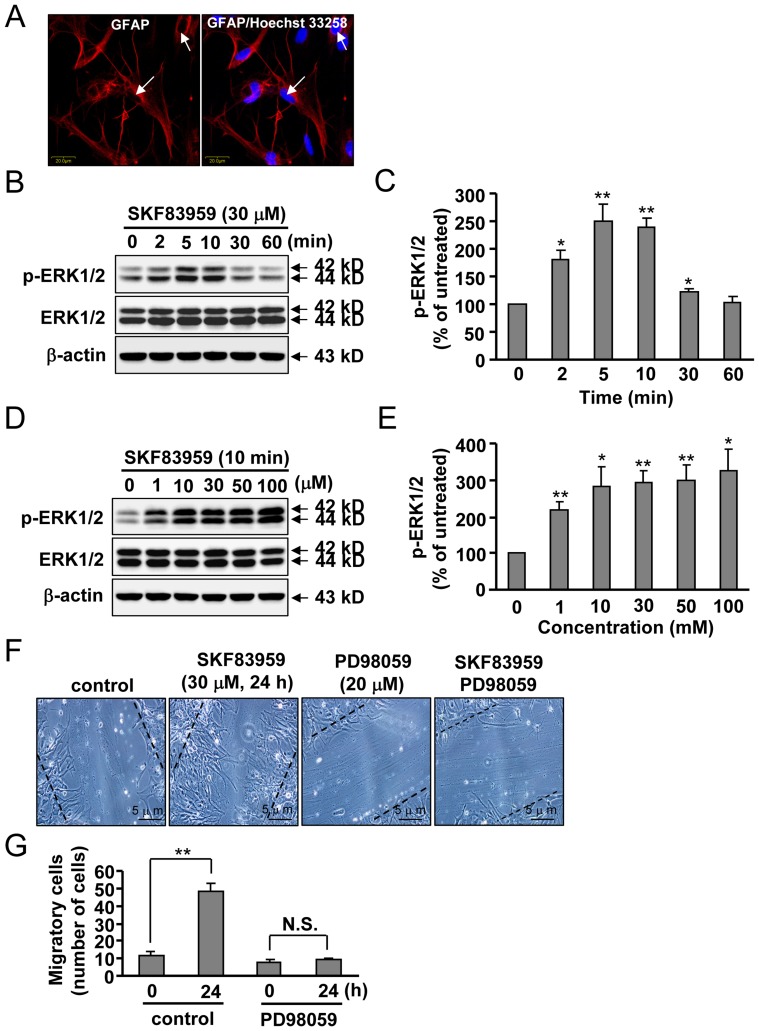
Effects of SKF83959 on ERK1/2 phosphorylation and cell migration in cultured rat astrocytes. (A) Cultured cortical astrocytes were identified using GFAP immunofluorescence and Hoechst 33258 staining. Bar: 20 µm. (B) Cultured astrocytes were incubated with 30 µM SKF83959 for 2 to 60 min. (C) A time-course analysis of ERK1/2 phosphorylation levels upon SKF83959 incubation from three independent experiments (Mean ± S.E., n = 3, **P < 0.01 or *P < 0.05, vs. control). (D) Cultured cortical astrocytes were treated with SKF83959 at different concentrations (1, 10, 30, 50, and 100 µM) for 10 min. (E) Quantification of ERK1/2 phosphorylation level in astrocytes upon incubation with different concentrations of SKF83959 in three independent experiments (Mean ± S.E., n = 3, **P < 0.01 or *P < 0.05, vs. control). (F) Effects of PD98059 (20 µM, 30 min), a specific inhibitor of MAP kinase, in SKF83959 (30 µM, 24 h)-induced migration of astrocytes. Bar: 5 µm. (G) Quantification of SKF83959-induced migration of astrocytes upon pretreatment with PD98059 (Mean ± S.E., n = 3, **P < 0.01, 24 h vs. 0 h). N.S.: no significance.

### Statistical Analysis

Data were expressed as mean ± SE. Comparisons for dose- and time-dependent data were made using a two-tailed Student' paired *t* test and corrected by Bonferroni. Comparisons for other data were made using unpaired *t* test. Differences were considered statistically significant at *P* < 0.05 or *P* < 0.01.

## Results

### SKF83959 Enhances ERK1/2 Phosphorylation and Cell Migration in Cultured Rat Astrocytes

Dopamine receptors have been reported to mediate ERK1/2 phosphorylation and activation in the nervous system. To evaluate the effects of the agonist of the putative PI-linked D_1_-like receptors SKF83959 on astrocytes, we first confirmed the purity of cultured cortical astrocytes. As shown in Fig. 1A, cell nuclei were stained with Hoechst 33258 and astrocytes were labeled with glial fibrillary acidic protein (GFAP), a marker specific to astrocytes, using immunofluorescence (purity > 98%, Fig. 1A). Purified astrocytes were treated with SKF83959 or vehicle for 2–60 min at concentrations ranging from 1–100 µM. ERK1/2 phosphorylation levels were measured by Western blot. As shown in Fig. 1B and 1C, SKF83959 induced a transient increase in ERK1/2 phosphorylation level in cultured astrocytes. Peak change was observed between 5 and 10 min after the application of SKF83959, and levels began to return to baseline at 30 min. A dose-dependent response curve showed that ERK1/2 phosphorylation became saturated at concentrations of 10, 30, and 50 µM SKF83959 and reached a maximum at 100 µM (Fig. 1D, 1E). For this reason, 10 min and 30 µM of SKF83959 incubation was selected for use in the following experiments. Because ERK1/2 affects astrocyte migration [18], [24], we evaluated the effects of SKF83959 on astrocyte migration. After 24 h of incubation, SKF83959 (30 µM) was found to significantly enhance cell migration (Fig. 1F, 1G). This effect was markedly inhibited by pretreatment with PD98059 (20 µM, 30 min), a specific inhibitor of mitogen-activated protein kinase kinase (Fig. 1F, 1G).

**Figure 2:**
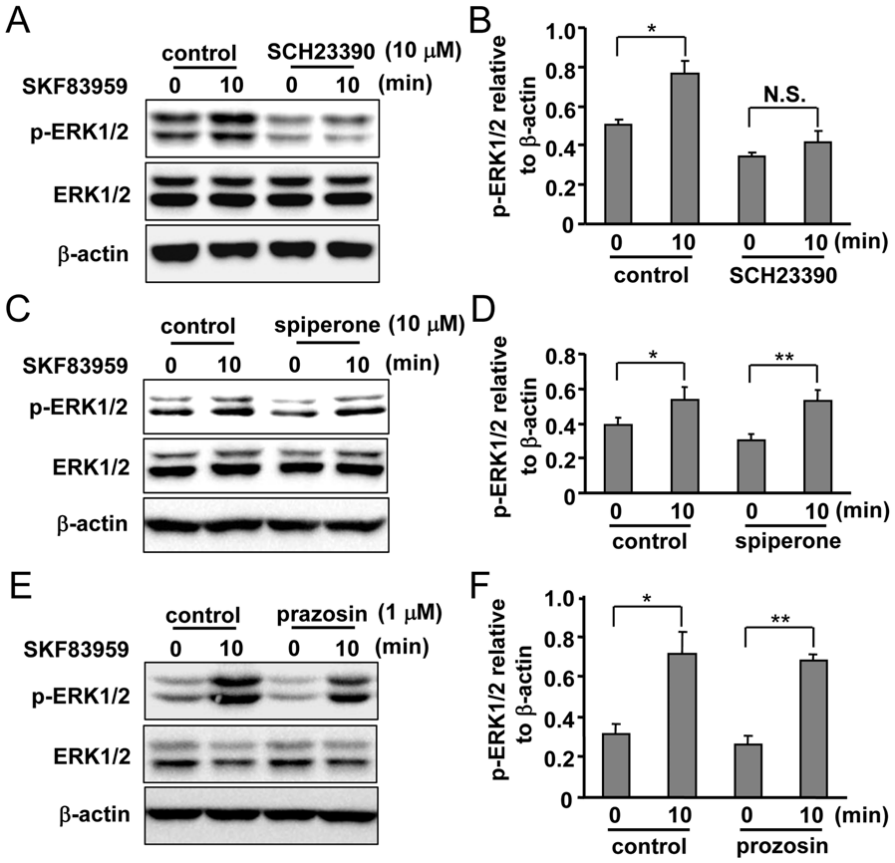
Involvement of putative PI-linked D_1_-like receptors in SKF83959-induced ERK1/2 phosphorylation. (A) Effects of D_1_ dopamine receptor antagonist SCH23390 (10 µM, 30 min) on SKF83959 (30 µM, 10 min)-induced ERK1/2 phosphorylation. (B) Quantification of ERK1/2 phosphorylation level in astrocytes pre-incubated with SCH23390 and treated with SKF83959 (Mean ± S.E., n = 3, *P < 0.05, 10 min vs. 0 min). N.S.: no significance. (C) Effects of D_2_ receptor antagonist spiperone (10 µM, 30 min) on SKF83959 (30 µM, 10 min)-induced ERK1/2 phosphorylation. (D) Quantification of ERK1/2 phosphorylation level in astrocytes pre-incubated with spiperone and treated with SKF83959 (Mean ± S.E., n = 3, **P < 0.01 or *P < 0.05, 10 min vs. 0 min). (E) Effects of α-adrenoceptor antagonist prazosin (1 µM, 30 min) on SKF83959 (30 µM, 10 min)-induced ERK1/2 phosphorylation. (F) Quantification of ERK1/2 phosphorylation level in astrocytes pre-incubated with prazosin and treated with SKF83959 (Mean ± S.E., n = 3, **P < 0.01 or *P < 0.05, 10 min vs. 0 min).

### Astrocytic PI-linked D_1_-like Receptors Mediate SKF83959-induced Increase in ERK1/2 Phosphorylation

Putative PI-linked D_1_-like receptors are the functional receptors for SKF83959. We next determined whether this receptor could mediate SKF83959-induced ERK1/2 phosphorylation. As expected, pretreatment of astrocytes with the selective antagonist of D_1_-like receptor SCH23390 (10 µM, 30 min) prior to SKF83959 (30 µM) incubation prevented increases in ERK1/2 phosphorylation (Fig. 2A, 2B). SKF83959 has a high affinity to D_1_-like receptor, but it also exhibits a weak or moderate affinity to other PLC-linked neurotransmitter receptors, such as D_2_ receptor and α-adrenoceptor. The roles of other receptors in SKF83959-induced ERK1/2 phosphorylation were then tested. Pre-incubation with an antagonist of D_2_ receptor spiperone (10 µM) (Fig. 2C, 2D) or an antagonist of α-adrenoceptor prazosin (1 µM) (Fig. 2E, 2F) did not exert inhibitory effects on SKF83959-induced ERK1/2 phosphorylation, suggesting that SKF83959-induced enhancement of ERK1/2 phosphorylation was mediated by neither the D_2_ receptor nor the α-adrenoceptor. In general, these results indicate that selective activation of the putative PI-linked D_1_-like receptors by SKF83959 is responsible for the increase in ERK1/2 phosphorylation.

**Figure 3:**
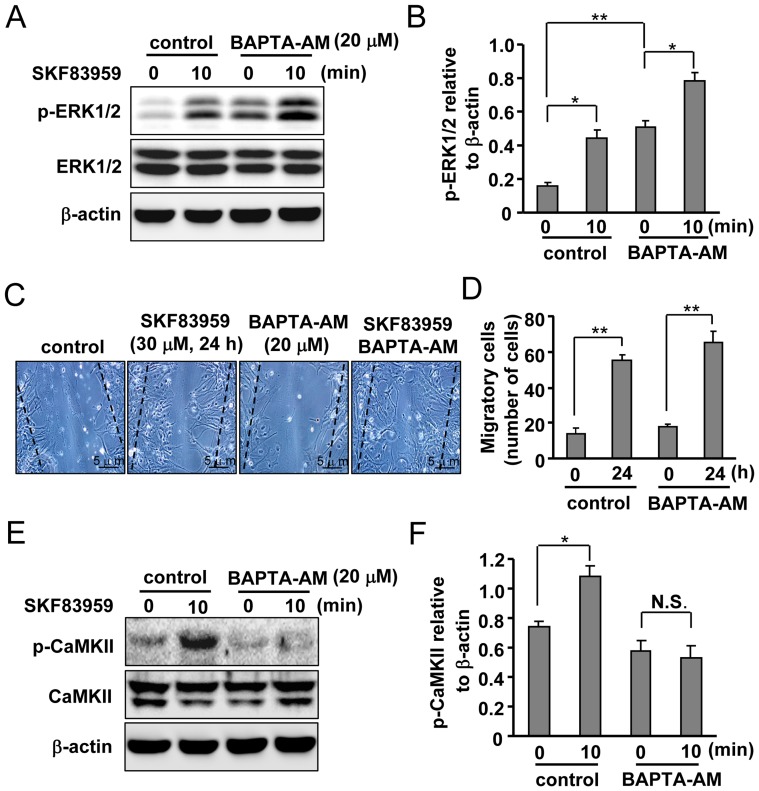
Contribution of intracellular Ca^2+^ to SKF83959-induced ERK1/2 phosphorylation. (A) Effects of BAPTA-AM (20 µM, 30 min) on SKF83959 (30 µM, 10 min)-induced ERK1/2 phosphorylation. (B) Quantification of ERK1/2 phosphorylation level in astrocytes pre-incubated with BAPTA-AM and treated with SKF83959 (Mean ± S.E., n = 3, *P < 0.05, 10 min vs. 0 min, **P < 0.01, BAPTA-AM 0 min vs. control 0 min). (C) Roles of BAPTA-AM (20 µM, 30 min) in SKF83959 (30 µM, 24 h)-induced migration of astrocytes. Bar: 5 µm. (D) Quantification of migration in astrocytes pre-incubated with BAPTA-AM and treated with SKF83959 (Mean ± S.E., n = 3, **P < 0.01, 24 h vs. 0 h). (E) Effects of BAPTA-AM (20 µM, 30 min) on SKF83959 (30 µM, 10 min)-induced CaMKII phosphorylation. (F) Quantification of CaMKII phosphorylation level in astrocytes pre-incubated with BAPTA-AM and treated with SKF83959 (Mean ± S.E., n = 3, *P < 0.05, 10 min vs. 0 min). N.S.: no significance.

### SKF83959-induced Increase in ERK1/2 Phosphorylation is Dependent on PLC but not on IP3/Ca^2+^ Signaling

The results of our previous study showed that SKF83959-induced activation of D_1_-like receptors increases intracellular Ca^2+^ level through mobilization of ER [15]. Because increases in internal Ca^2+^ can trigger ERK1/2 phosphorylation, we investigated whether an increase in internal Ca^2+^ could mediate SKF83959-induced ERK1/2 phosphorylation. Astrocytes were pretreated with a Ca^2+^ chelator BAPTA-AM (20 µM, 30 min), followed by application of SKF83959 (30 µM) for 10 min. BAPTA-AM failed to prevent the SKF83959-induced increase in ERK1/2 phosphorylation level, demonstrating that increases in intracellular Ca^2+^ are not necessary for the elevation in ERK1/2 phosphorylation level (Fig. 3A, 3B). Pretreatment with BAPTA-AM also failed to inhibit astrocyte migration, indicating that SKF83959 (24 h)-induced astrocyte migration is also not dependent on increases in internal Ca^2+^ (Fig. 3C, 3D). In addition, BAPTA-AM itself elevated the basal ERK1/2 phosphorylation level of cultured astrocytes.

**Figure 4:**
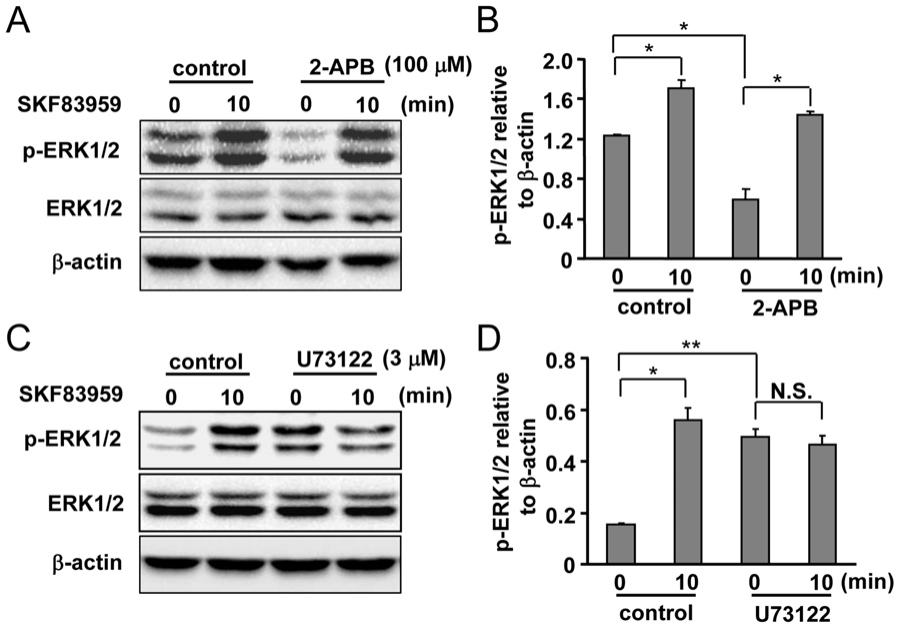
Roles of PLC and IP3 receptor in the mediation of SKF83959-induced ERK1/2 phosphorylation. (A) Representative Western blot results indicating the effects of 2-APB (100 µM, 30 min) on SKF83959 (30 µM, 10 min)-induced ERK1/2 phosphorylation. (B) Quantitative analysis of pretreatment with 2-APB on SKF83959-induced ERK1/2 phosphorylation (Mean ± S.E., n = 3, *P < 0.05, 10 min vs. 0 min, *P < 0.05, 2-APB 0 min vs. control 0 min). (C) Representative Western blot results showing the effects of U73122 (3 µM, 30 min) on SKF83959 (30 µM, 10 min)-induced ERK1/2 phosphorylation. (D) Quantitative analysis of pretreatment with U73122 on SKF83959-induced ERK1/2 phosphorylation (Mean ± S.E., n = 3, *P < 0.05, 10 min vs. 0 min, **P < 0.01, U73122 0 min vs. control 0 min). N.S.: no significance.

**Figure 5:**
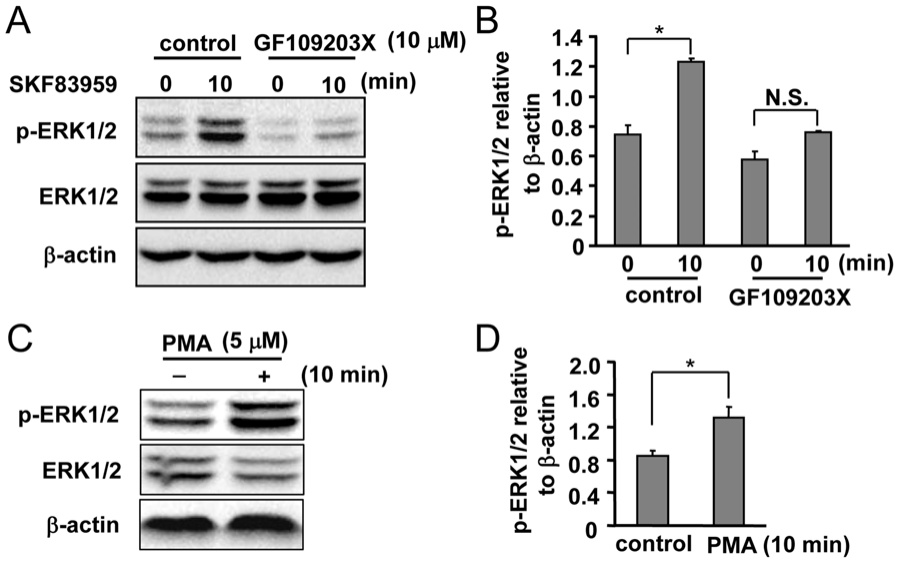
Role of PKC in SKF83959-induced ERK1/2 phosphorylation. (A) Effects of the non-selective PKC inhibitor GF109203X (10 µM, 30 min) on SKF83959 (30 µM, 10 min)-induced ERK1/2 phosphorylation. (B) Quantitative analysis of pretreatment with GF109203X on SKF83959-induced ERK1/2 phosphorylation (Mean ± S.E., n = 3, *P < 0.05, 10 min vs. 0 min). N.S.: no significance. (C) Effects of the specific PKC activator PMA (5 µM, 10 min) on ERK1/2 phosphorylation in cultured astrocytes. (D) Quantification of ERK1/2 phosphorylation level in astrocytes incubated with PMA (5 µM, 10 min) (Mean ± S.E., n = 3, *P < 0.05, vs. control).

**Figure 6:**
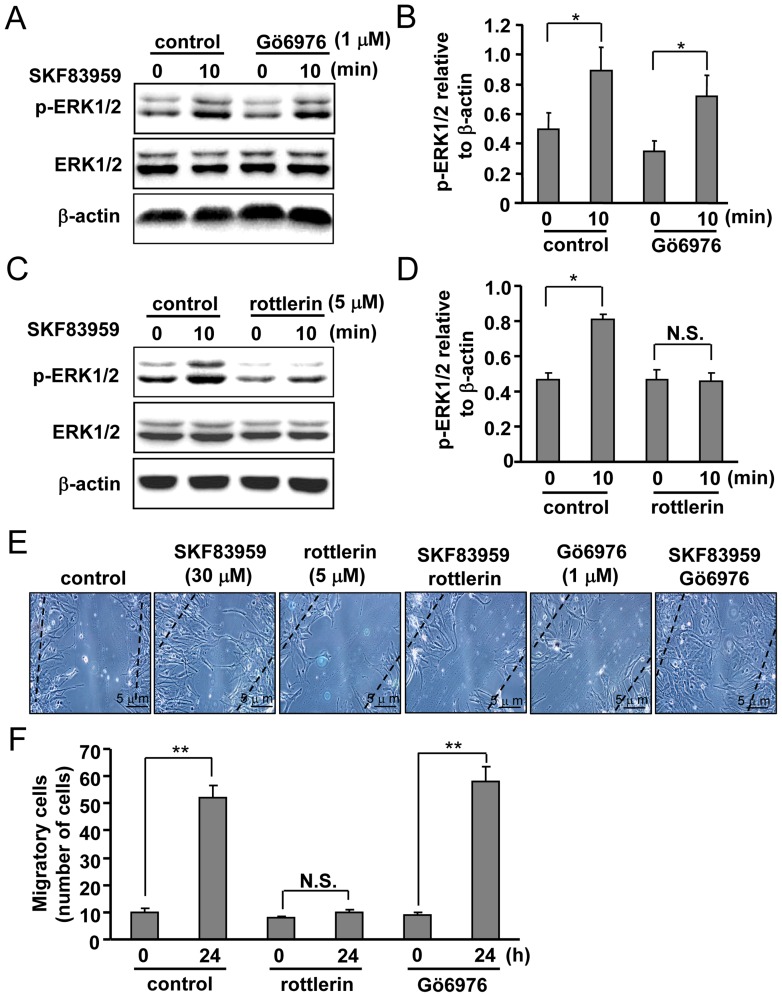
Roles of PKCδ and PKCα in SKF83959-induced ERK1/2 phosphorylation. (A) Effects of the PKCα inhibitor Gö6976 (1 µM, 30 min) on SKF83959 (30 µM, 10 min)-induced ERK1/2 phosphorylation. (B) Quantitative analysis of pretreatment with 1 µM Gö6976 on SKF83959-induced ERK1/2 phosphorylation (Mean ± S.E., n = 3, *P < 0.05, 10 min vs. 0 min). (C) Effects of PKCδ inhibitor rottlerin (5 µM, 30 min) on SKF83959 (30 µM, 10 min)-induced ERK1/2 phosphorylation. (D) Quantitative analysis of pretreatment with rottlerin on SKF83959-induced ERK1/2 phosphorylation (Mean ± S.E., n = 3, *P < 0.05, 10 min vs. 0 min). N.S.: no significance. (E) Respective roles of rottlerin (middle, 5 µM, 30 min) and Gö6976 (right, 1 µM, 30 min) in SKF83959 (30 µM, 24 h)-induced migration of astrocytes. Bar: 5 µm. (F) Statistical analysis of respective pretreatment with rottlerin (middle) and Gö6976 (right) on SKF83959-induced migration of astrocytes (Mean ± S.E., n = 3, **P < 0.01, 24 h vs. 0 h). N.S.: no significance.

Next, to confirm the specificity of BAPTA-AM as a Ca^2+^ chelator, we selected a well-known Ca^2+^/calmodulin binding protein CaMKII [25], [26] as a positive control and evaluated SKF83959-induced CaMKII phosphorylation in the absence or presence of BAPTA-AM. As anticipated, SKF83959-induced enhancement of CaMKII phosphorylation was almost completely abolished by pretreatment with BAPTA-AM (20 µM, 30 min), suggesting that intracellular Ca^2+^ was indeed chelated by BAPTA-AM (Fig. 3E, 3F). To further confirm the contribution of intracellular Ca^2+^ on SKF83959-induced ERK1/2 phosphorylation increase, changes in ERK1/2 phosphorylation were assayed upon application of 2-APB, a blocker of ER IP3 receptors. As shown in Fig. 4A and 4B, 2-APB (100 µM) did not affect ERK1/2 phosphorylation level either. Surprisingly, we noted that 2-APB itself significantly reduced the basal ERK1/2 phosphorylation level of cultured astrocytes (Fig. 4A, 4B).

Because BAPTA-AM and 2-APB did not inhibit ERK1/2 phosphorylation induced by SKF83959, we assessed the effects of PLC, an upstream molecule of the mobilization of intracellular Ca^2+^, on SKF83959-induced ERK1/2 phosphorylation. U73122, a specific inhibitor of PLC, counteracted SKF83959’s (30 µM, 10 min) ability to elevate the level of ERK1/2 phosphorylation (Fig. 4C, 4D). This indicates that other IP3-independent signaling pathways might contribute to SKF83959-induced ERK1/2 phosphorylation in our current system. A marked elevation in the basal level of ERK1/2 phosphorylation was observed after U73122 treatment in cultured astrocytes, like BAPTA-AM treatment (Fig. 4C, 4D).

### PKCδ is Necessary for SKF83959-induced Increase in ERK1/2 Phosphorylation and Cell Migration in Cultured Rat Astrocytes

PKC is one of the molecules downstream of PLC activation. We determined whether SKF83959-induced ERK1/2 phosphorylation is involved in PKC activation. As shown in Fig. 5A and 5B, the non-selective PKC inhibitor GF109203X (10 µM) significantly inhibited the increase in ERK1/2 phosphorylation level induced by SKF83959. To confirm the involvement of PKC in astrocytic ERK1/2 phosphorylation, astrocytes were incubated with PMA (5 µM), a specific activator of PKC. Our data show that PMA successfully enhanced ERK1/2 phosphorylation in cultured astrocytes, suggesting that PKC is indeed involved in SKF83959-induced ERK1/2 phosphorylation (Fig. 5C, 5D).

PKC exists in various isoforms with different characteristics. For example, activation of the typical PKCα isoform is dependent on both Ca^2+^ and DAG, but activation of the novel PKCδ isoform is dependent on DAG but not on Ca^2+^. We therefore investigated the roles of PKC isoforms in SKF83959-induced ERK1/2 phosphorylation using various pharmacological inhibitors. Rottlerin (5 µM), a specific inhibitor of PKCδ, significantly reduced the SKF83959-induced increase in ERK1/2 phosphorylation, and the PKCα inhibitor Gö6976 (1 µM) did not (Fig. 6A, 6B, 6C, 6D). In line with the effect of rottlerin on SKF83959-induced ERK1/2 phosphorylation, rottlerin markedly attenuated SKF83959 (24 h incubation)-induced astrocyte migration (Fig. 6E, 6F). Pretreatment with Gö6976 (1 µM, 30 min) had no effect on SKF83959-induced astrocyte migration (Fig. 6E, 6F). In conclusion, these data indicate that PKCδ functionally mediates the increase in ERK1/2 phosphorylation induced by SKF83959 in cultured rat astrocytes.

**Figure 7:**
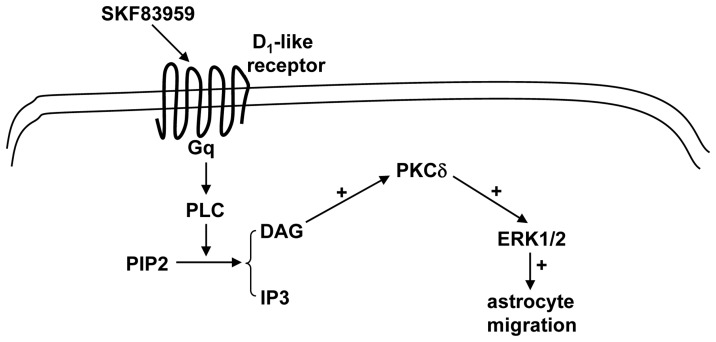
Proposed scheme for the signaling pathways downstream of SKF83959-induced ERK1/2 phosphorylation in cultured rat astrocytes.

## Discussion

The classical DA receptor subtypes are widely expressed in astrocytes [13], [14]. However, the functional roles of the putative PI-linked D_1_-like receptors in these cells are still not well understood. In the present study, we demonstrate that SKF83959 evokes a significant elevation in ERK1/2 phosphorylation in a time- and dose-dependent manner in cultured rat astrocytes. This elevation was transient and returned to the baseline within 60 min. However, one previous study demonstrated that it was CaMKII and CDK5, not ERK1/2, that was activated by SKF83959 [10]. The difference was probably due to the different samples used: SKF83959 was applied into cultured astrocytes in our current study, but the previous study was performed on brain slices [10]. SKF83959 is a specific agonist of the putative PI-linked D_1_-like receptors and SCH23390, D_1_-like receptor antagonist, was indeed found to prevent SKF83959-induced ERK1/2 phosphorylation elevation. The fact that the antagonists of D_2_ receptor and α-adrenoceptor both failed to inhibit ERK1/2 phosphorylation indicates that SKF83959-induced ERK1/2 phosphorylation involves putative PI-linked D_1_-like receptors.

ERK1/2 phosphorylation elevates ERK1/2 activity, which then promotes astrocyte migration [24]. Astrocytes are involved in various pathological conditions through membrane receptors such as the N-methyl-D-aspartic acid (NMDA) receptors and classical DA receptors [14], [27]. Astrocytic putative PI-linked D_1_-like receptors were also reported to participate in internal Ca^2+^ mobilization and FGF-2 production in astrocytes [6]. Hence, we speculate that SKF83959 might influence astrocyte migration by regulating ERK1/2 phosphorylation. As shown in Fig. 1F and 1G, SKF83959 significantly promoted astrocyte migration, which could be largely inhibited by ERK1/2 inhibition. In this way, our results show that SKF83959 promoted ERK1/2 activation and the subsequent astrocyte migration, at least under *in vitro* conditions.

A number of studies have confirmed that the putative PI-linked D_1_-like receptors are Gq-related. Upon coupling to Gq protein, putative PI-linked D_1_-like receptors promoted IP3 production and the subsequent release of internal Ca^2+^ [28]. Although elevated levels of internal Ca^2+^ might mediate the activation of kinases such as CaMKII and ERK1/2 [10], [29], BAPTA-AM-mediated chelation of intracellular Ca^2+^ did not inhibit SKF83959-induced ERK1/2 phosphorylation. However, as a positive control, the phosphorylation of classical Ca^2+^/camodulin-regulated kinase CaMKII was almost totally abolished by BAPTA-AM pretreatment [25], [26]. In this way, these data indicate that the effects of SKF83959 on ERK1/2 activation are probably Ca^2+^-independent. This conclusion is further supported by the failure of 2-APB to inhibit SKF83959-induced ERK1/2 phosphorylation. However, other data are inconsistent with a Ca^2+^-independent scenario. BAPTA-AM itself markedly increased the basal level of ERK1/2 phosphorylation, and 2-APB reduced it, suggesting that Ca^2+^ might negatively regulate ERK1/2 activation. One previous study reported that calcineurin physically interacted with ERK1/2 and restrained its activity through de-phosphorylation [30]. Because Ca^2+^ generally binds to calcineurin, we postulated that BAPTA-AM itself might enhance ERK1/2 phosphorylation by eliminating negative Ca^2+^-calcineurin signals [31]. The compensatory Ca^2+^ entrance after 2-APB treatment might contribute to the ability of 2-APB to cause these reductions.

PLC is the pivotal molecule responsible for IP3 production [28]. We used a PLC inhibitor to investigate the role of PLC in SKF83959-induced ERK1/2 phosphorylation. Astrocytes exposed to the PLC inhibitor exhibited a significant reduction in the level of ERK1/2 phosphorylation, demonstrating that other PLC-associated signals might be involved in SKF83959-induced ERK1/2 phosphorylation. As with BAPTA-AM’s effect on ERK1/2 phosphorylation level, we observed an elevation in basal ERK1/2 phosphorylation level after treatment with U73122 alone. PLC activation causes increases in intracellular Ca^2+^ through two pathways, ER mobilization, and Ca^2+^ influx after attenuation of PIP2’s inhibition of membrane ligand-gated ion channels [32], [33]. The reduction of Ca^2+^ influx after U73122 treatment might explain the increase in the basal level of ERK1/2 phosphorylation. Further investigations are required to evaluate the function of Ca^2+^-related signals in ERK1/2 activation in physiological conditions.

Diacylglycerol (DAG) is also present in the cytosol after PLC activation. DAG couples to PKC and positively regulates PKC activity in various disorders such as diabetes and cancer [34], [35]. In our study, a non-selective PKC inhibitor effectively inhibited SKF83959-induced increases in ERK1/2 phosphorylation. Generally, PKC is divided into three sub-groups: conventional PKCs (α, βI, βII, and γ); novel PKCs (δ, ε, η, and θ); and atypical PKCs (ζ and λ) [36]. Distinct PKC isoforms take different characteristics [37]. For example, the conventional PKCs are Ca^2+^-dependent and activated by DAG, but novel PKCs are Ca^2+^-independent. The atypical PKCs are both Ca^2+^- and DAG-independent. To determine which specific isoform is involved in SKF83959-induced ERK1/2 phosphorylation, we investigated the effects of the different PKC isoforms on ERK1/2 phosphorylation. We found that PKCα is not involved in ERK1/2 phosphorylation, but PKCδ inhibition robustly attenuated SKF83959-induced ERK1/2 phosphorylation and astrocyte migration. This is in accordance with the role of PKCδ in bradykinin-induced ERK1/2 phosphorylation and astrocyte migration [24]. It may provide us with a new target for the determination of the function of SKF83959.

In conclusion, our current data clearly suggest that the PLC-DAG-PKCδ signaling pathway mediates the SKF83959-induced ERK1/2 activation in cultured rat astrocytes. This might help us to understand the new pharmacological roles of SKF83959 in the nervous system (Fig. 7).
